# Behavioral trait (co)variances and plasticity in response to turbidity in wild zebrafish (*Danio rerio*)

**DOI:** 10.1242/bio.062341

**Published:** 2026-02-24

**Authors:** Bhavya Pratap Singh, Anuradha Bhat

**Affiliations:** Department of Biological Sciences, Indian Institute of Science Education and Research (IISER) Kolkata, Mohanpur – 741246, West Bengal, India

**Keywords:** Trait (co)variances, Correlated behavioral plasticity, Coping behaviors, MCMCglmm, Multivariate mixed modeling, Turbid waters, Zebrafish

## Abstract

The unpredictability of the environment can shape not only the mean behavioral expression but also the structure of behavioral variance within wild populations. Yet, empirical tests integrating individual variation, trait covariances, and correlated plasticities across ecologically relevant gradients remain rare, particularly those aimed at phenotype integration. Using wild zebrafish, we examined how long-term exposure to turbidity and immediate changes in water clarity influence behavioral means, individual (co)variances, and plasticity integration across three coping behaviors – activity, aggression, and boldness. Adult fish were conditioned in clear and turbid water for a month, followed by repeated behavioral tests in clear as well as in turbid water. Individuals that were maintained in clear or turbid water for a month showed substantial variation in their behavioral adjustments when exposed to perturbations in the water-clarity. Mean behaviors changed primarily in response to immediate change in water turbidity and trial repetition, not long-term conditioning. However, conditioning strongly altered individual variance structure; turbid-conditioned individuals showed reduced consistency across testing waters, indicating greater behavioral flexibility under sensory uncertainty. The only evidence to behavioral syndromes was between activity and boldness [r=−0.379] among clear-conditioned treatment, whereas within-individual correlations between aggression and boldness were prominent in turbid-conditioned fish. Despite substantial variation in behavioral plasticity, we detected no among-individual correlations in plasticity across traits. Together, these results demonstrate that turbidity modulates multi-scale behavioral variation, influencing how traits covary and integrate within individuals, and highlight how environmental unpredictability shapes flexibility in coping strategies.

## Teaser

**Summary:** Environmental unpredictability affects behavioral trait covariation, and within-individual integration, enhancing plasticity and flexibility in zebrafish coping behaviors.

## INTRODUCTION

Understanding how animals respond to environmental variation requires examining not only behavioral means but also how traits vary and covary at multiple hierarchical levels (Armbruster et al., 2014; Westneat et al., 2015). Insights from studies on consistent patterns of behavioral variation or covariation among suites of behaviors across time and context have led to the concept of ‘animal personality’ and ‘behavioral syndromes’ in wild populations (Carter et al., 2013; Kaiser and Müller, 2021; Sih et al., 2004; Briffa and Weiss, 2010). However, trait (co)variances (Hadfield, 2010; Dingemanse and Dochtermann, 2013) may not be strictly consistent in changing environments (Mitchell and Houslay, 2021), implying that the trait (co)variation is in tandem with behavioral plasticity (Dingemanse et al., 2010; Ellers and Liefting, 2015). Despite being extensively linked, so far empirical work on trait covariances and behavioral plasticity has been independent of each other; plasticity analyses focus on a single trait across an environment gradient while trait covariances consider correlations among multiple traits in an environmental regime. Mitchell and Houslay (2021) suggested the unification of these two frameworks for the understanding of how traits are constrained or integrated in a coordinated fashion in response to environmental changes. The appropriate statistical rigor to achieve this has become possible with the recent advances in multivariate regression analyses.

**Fig. 1.**
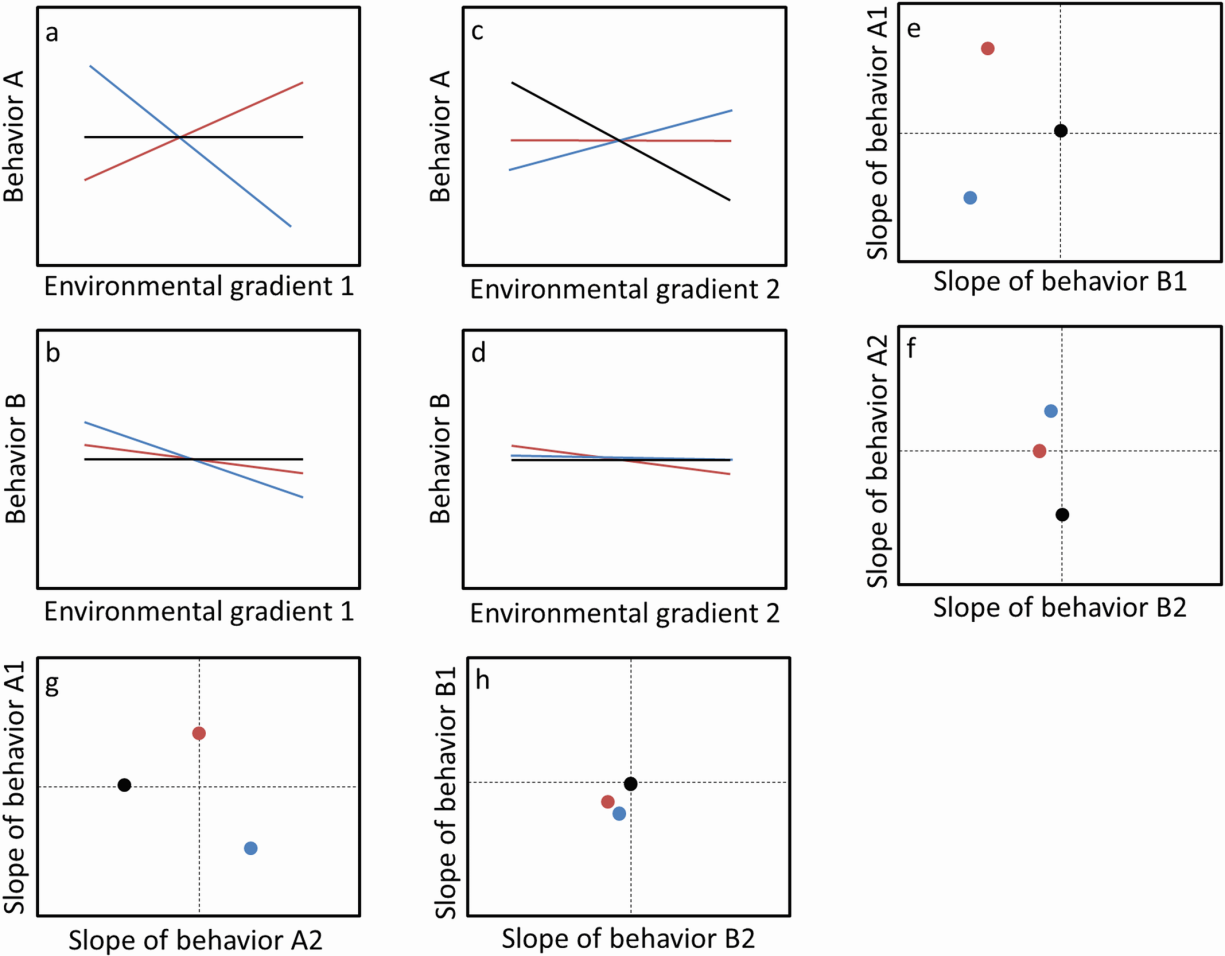
**A hypothetical illustration of correlated behavioral plasticities among three individuals (black, blue and red).** Behavior A and behavior B across two different environmental gradients (represented as A1, A2 and B1, B2) such as food availability and the complexity of the environment (A,B,C,D). Among-behaviors slope (E,F) does not have any obvious correlation pattern between slope direction; similar to within-behaviors slope pattern (G,H) (adopted from Sheehy and Laskowski, 2023).

In a broader sense, behavioral plasticity refers to the average change in an individual's response across an environmental gradient (Kelly et al., 2012; Stamps and Biro, 2016; Stamps, 2016). A simpler way to represent this within-individual variation across environmental gradients is via behavioral reaction norm (BRN) (Dingemanse et al., 2010). Since genotypes or individuals differ in their levels of behavioral plasticity, intercepts of BRNs represent the among-individual variation in a behavioral trait while the slopes of BRNs represent the among-individual variation in behavioral plasticity across a focal environmental gradient. The induction of within-individual variation in behavior could be in response to external stimuli (described as exogenous plasticity) or spontaneous fluctuations in the internal state (described as endogenous plasticity). Plasticity can also differ in the temporal scale of environment influence; contextual plasticity describes responses to current conditions, whereas experiential or developmental plasticity reflects the effects of past experiences (Stamps and Groothuis, 2010; Snell-Rood, 2013; Urszán et al., 2018). Such distinctions in behavioral plasticity are necessary to determine whether some genotypes/individuals are, in general, more plastic than others, and can be established by quantifying the correlations between these distinctive plasticities (Stamps, 2016). Because behavioral responses often involve multiple traits, such correlations may occur within and among behaviors. We refer to correlations among different forms of plasticity within a single trait as within-behavior correlated plasticities, and correlations in the same type of plasticity across traits as among-behavior correlated plasticities. Together, these forms constitute plasticity integration (Fig. 1; Stamps, 2016; Sheehy and Laskowski, 2023).

Aquatic environments offer an ideal system for testing these ideas because of their dynamic nature and imposed recurrent environmental challenges (Soukup et al., 2022). Animals often exhibit a coherent set of behavioral and physiological stress responses against any environmental stressors, giving a general adaptive pattern to everyday challenges in an animal's natural habitat, also known as coping styles. Patterns in vertebrate systems suggest two broad coping styles: proactive, where individuals are consistently active, aggressive, bold, explorative, and show lesser degree of plasticity; and reactive, where individuals are less-active, less-aggressive, shier, less-explorative and show higher degrees of plasticity (Koolhaas et al., 1999, 2007; Øverli et al., 2007; Koolhaas, 2008). While there is enough evidence for the existence of these coping styles, the generalities of them across taxa is still debatable. So far, what we understand is that coping strategies exhibit context-dependency (Stamps and Groothius, 2010) and the varying degrees of coping strategies among individuals against varied environmental stressors can have important evolutionary and ecological implications (Øverli et al., 2007; Sih et al., 2012; Wolf and Weissing, 2012; Mathot and Dingemanse, 2015). Fish species have been extensively used as models to investigate coping-styles against environmental stressors such as presence of predators, rise in temperature, space-confinement, etc. (Vindas et al., 2017). One such stressor of interest is change in water clarity, due to rise in turbidity. Suspended particles in water can attenuate light due to their light-scattering properties, which makes water appear cloudier (Davies-Colley and Smith, 2001). Freshwater bodies face fluctuations in sediment-associated turbidity that can be due to natural causes like rainfall and flooding. However, anthropogenic drivers including agricultural runoff, deforestation, industrial effluents, and hydrological alterations have intensified the magnitude and duration of turbidity, with fine sediment inputs expected to rise further (Scheurer et al., 2009). Consequently, eutrophication and sedimentation are driving rapid changes to the sensory environment. Behaviors in aquatic organisms that are dependent on vision are known to be influenced by such rapid changes in water-light conditions (Gray et al., 2012a,b; Sweka and Hartman, 2001; Sekhar et al., 2019; Kelley et al., 2012; Abrahams and Kattenfeld, 1997; Sundin et al., 2010; Borner et al., 2015; Sohel and Lindström, 2015). There is, however, not enough understanding on the prolonged effect of turbidity (but see Suriyampola et al., 2018) on behavioral (co)variances, or the integration of plasticity to reshape trait integration altogether. For this study, we chose adult zebrafish as our model system, for which we quantified among- and within-individual variation in their three coping behaviors across two discrete environments using a multivariate reaction-norm approach. We then estimated correlations among traits and among plasticities to assess how environmental change shapes behavioral integration and the mechanisms underlying coping strategies.

Zebrafish (*Danio rerio*) (Hamilton, 1822) is a teleost fish, found in tropical freshwater streams and stagnant pool habitats of the Indian subcontinent (Engeszer et al., 2007; Spence et al., 2008). It is a popular model organism used in research in genetics, neuroscience, development, behavior, etc. (Sullivan and Kim, 2008; Norton and Bally-Cuif, 2010; MacRae and Peterson, 2015; Meyers, 2018; Chia et al., 2022). In zebrafish habitats, turbidity is often a transient condition due to the partial settling of suspended particles – unless the environment is frequently disturbed. Genotypes experiencing differing levels of turbidity in their early life (e.g. experienced in larval stages) may have canalized response to the rise in turbidity. While the immediate responses to environmental changes as an adult might be due to the cumulative effect of all the experiences (Stamps and Groothius, 2010). This raises an interesting question about the long-term and immediate effects of turbidity on their coping and risk-taking behaviors. Previous studies on the system have demonstrated consistency in individual variation in boldness, exploration, aggression, sociability and learning across different populations (Roy and Bhat, 2018a,b; Daniel and Bhat, 2022a). But to our knowledge, no study has examined trait (co)variances and correlated plasticities among traits in the context of turbidity. In line with the conceptual frameworks presented above, we asked the following questions: (1) do zebrafish predisposed to different levels of water-clarity have different adjusting responses in changing environments? We addressed this by conditioning individuals in clear and turbid water regimes for a month (following Suriyampola et al., 2018) and tested their behavioral traits – such as activity, aggression, and boldness – in both clear and turbid waters. We expected fish conditioned in turbid water to show reduced immediate behavioral flexibility when shifted to clear water, due to sensory habituation and a reduced reliance on visual cues in chronically turbid environments. (2) Can correlated behavioral plasticities explain the emergence of behavioral syndromes or vice versa? To investigate this, we analyzed (co)variance(s) in the behavioral phenotypes across different contexts and expected pronounced syndromes and correlated plasticities among clear-conditioned. Clear-conditioned fish, experiencing higher visual information and less sensory constraint, should exhibit stronger behavioral syndromes and more pronounced correlated plasticities (Sheehy and Laskowski, 2023). We also expected differences between sexes – females to be less-active, less-aggressive, and shier due to their reactive mating strategies (Spence et al., 2008; Owen et al., 2012).

## RESULTS

### Mean-effects

**Fig. 2.**
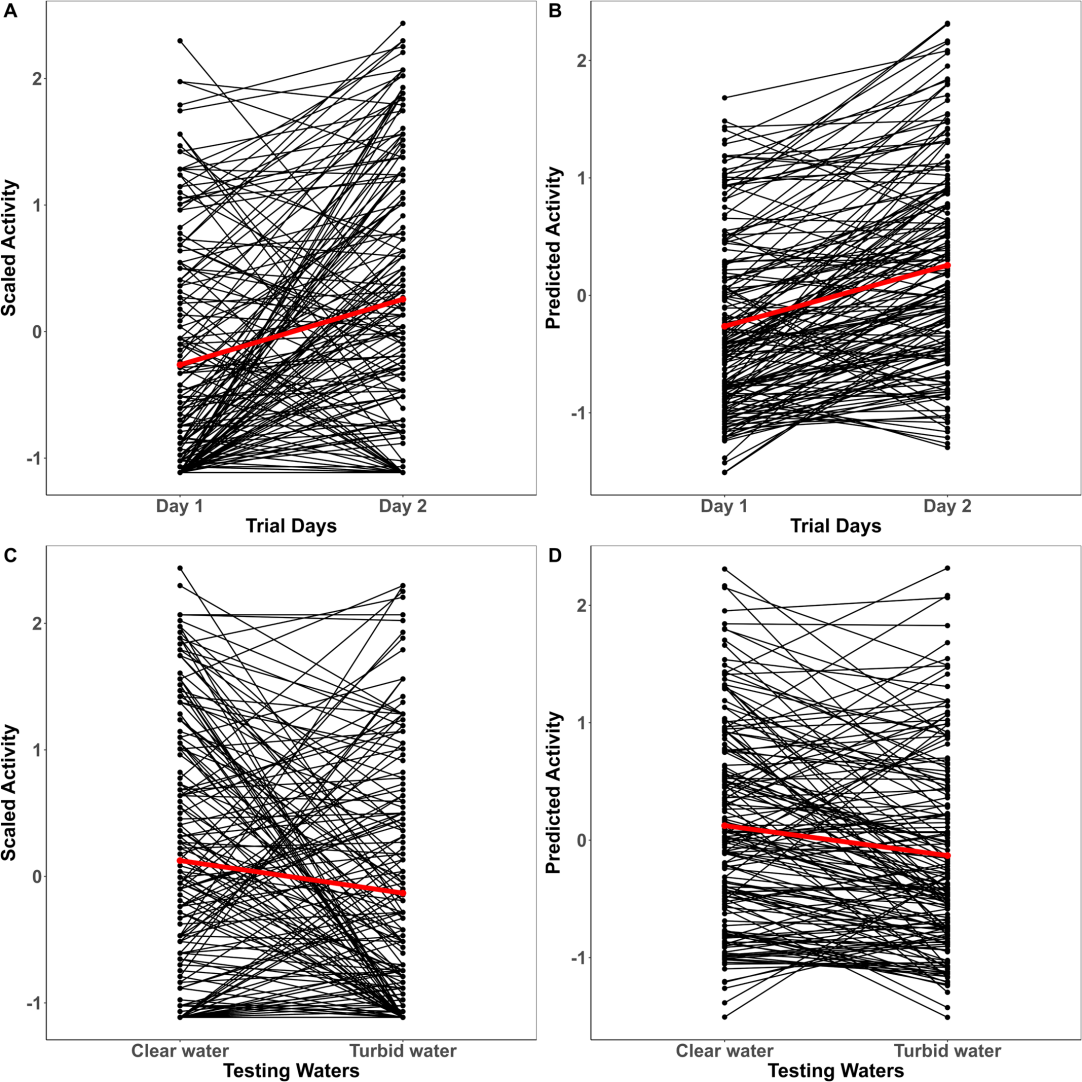
**Individual variation in activity across trial days (A,B) and testing waters (C,D) for conditioning regimes plotted together.** Black lines correspond to the individual-level reaction norms and red lines represent the mean-level trend. Z-transformed raw scores for activity are plotted in A and C, while their corresponding model predictions are shown in B and D.

**Fig. 3.**
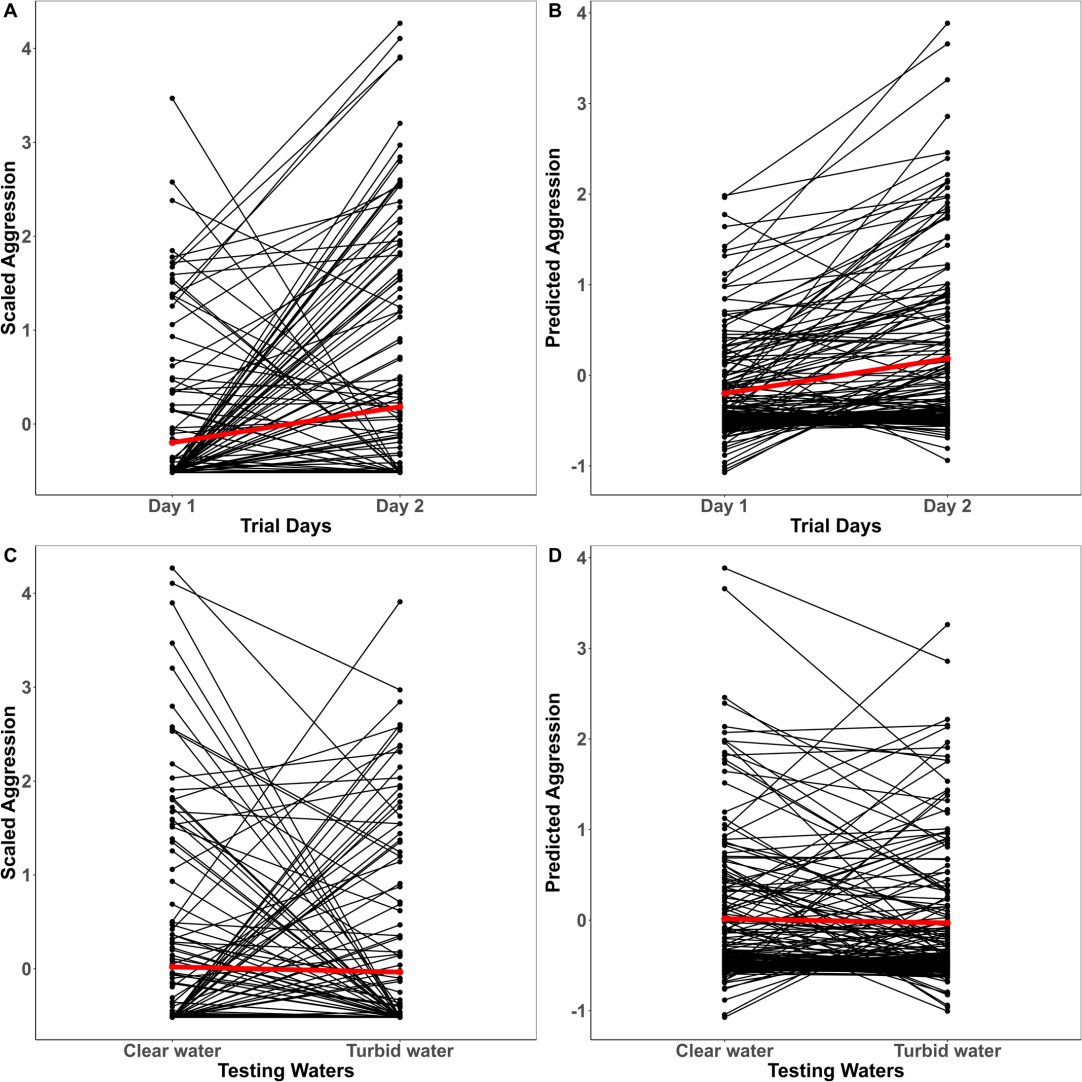
**Individual variation in aggression across trial days (A,B) and testing waters (C,D) for conditioning regimes plotted together.** Black lines correspond to the individual-level reaction norms and red lines represent the mean-level trend. Z-transformed raw scores for aggression are plotted in A and C, while their corresponding model predictions are shown in B and D.

**Fig. 4.**
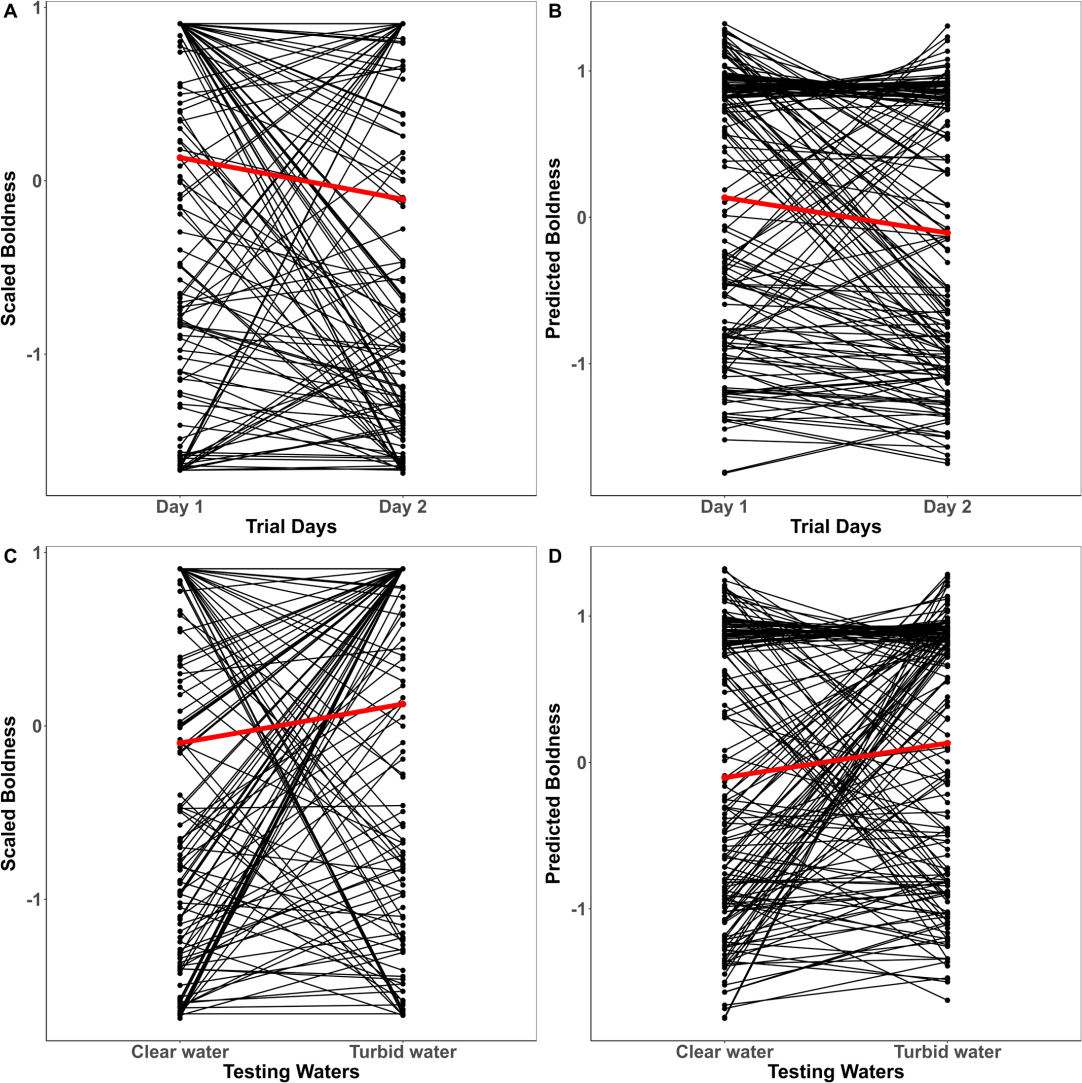
**Individual variation in boldness across trial days (A,B) and testing waters (C,D) for conditioning regimes plotted together.** Black lines correspond to the individual-level reaction norms and red lines represent the mean-level trend. Z-transformed raw scores for boldness are plotted in A and C, while their corresponding model predictions are shown in B and D.

There was no significant effect of conditioning on any of the measured traits (Table 1). In contrast, immediate change in water clarity decreased activity [−0.198 (−0.375, 0.008); pMCMC=0.042] from clear to turbid waters. Trial repetition had a significant effect on activity [0.578 (0.291, 0.838)] and aggression [0.303 (0.016, 0.630)] such that there was a slight increase in mean expression of traits from day one to day two (Figs 2 and 3). Though trends suggested slightly faster emergence on the second trial, the mean-shift in boldness, however, was not reflected in our model estimates [−0.130 (−0.279, 0.008); pMCMC=0.077] (Fig. 4, Table 1). There were significant trial days×testing waters interaction effects across all three traits indicating that temporal and contextual effects jointly shaped behavioral expression (Table 1).

**Table 1.** Parameter estimates (posterior means) for fixed-effects in activity, aggression and boldness, reported alongside the lower and upper bounds of 95% CI

	Activity Posterior mean (95% CI)	Aggression Posterior mean (95% CI)	Boldness Posterior mean (95% CI)
Intercept	**−2.354 [−4.266, −0.129]**	0.490 [−1.350, 2.390]	−0.506 [−1.592, 0.559]
Trial day	**0.578 [0.291, 0.838]**	**0.303 [0.016, 0.630]**	−0.130 [−0.279, 0.008]
Testing water	**−0.198 [−0.375, 0.008]**	−0.103 [−0.331, 0.093]	−0.045 [−0.179, 0.104]
Conditioning regime	0.130 [−0.194, 0.476]	0.010 [−0.253, 0.300]	−0.108 [−0.281, 0.073]
Trial day×testing water	**−0.102 [−0.237, 0.031]**	**0.151 [0.009, 0.297]**	**0.152 [0.049, 0.258]**
Trial×conditioning regime	−0.033 [−0.403, 0.322]	−0.022 [−0.418, 0.330]	−0.091 [−0.307, 0.094]
Nudged	−0.091 [-0.239, 0.062]	**−0.147 [−0.290, 0.008]**	**1.401 [1.302, 1.512]**
Size	**0.895 [0.053, 1.847]**	−0.263 [−1.046, 0.549]	0.013 [−0.428, 0.507]
Sex	**4.063 [0.942, 7.373]**	−0.673 [−3.326, 2.238]	−1.122 [−2.871, 0.697]
Size×sex	**−1.665 [−3.016, −0.257]**	0.312 [−0.807, 1.566]	0.466 [−0.355, 1.174]

Boldfaced estimates represent where the CIs did not overlap zero, indicating significance. The clear-conditioned regime, day 1, CW as testing conditions, female and ‘no’-nudged are the reference groups.

While nudging had a significant effect on boldness [1.401 (1.302, 1.512)], its effect on aggression [−0.147 (−0.290, 0.008)] and activity [−0.091 (−0.239, 0.062)] were not significant. Larger individuals were more active and homogeneous in their movements throughout the tank. Females and males also differed in their activity patterns; there was also a significant interaction effect between sex and size on activity (Table 1).

### Among individual variances and correlations in intercepts and slopes

**Fig. 5.**
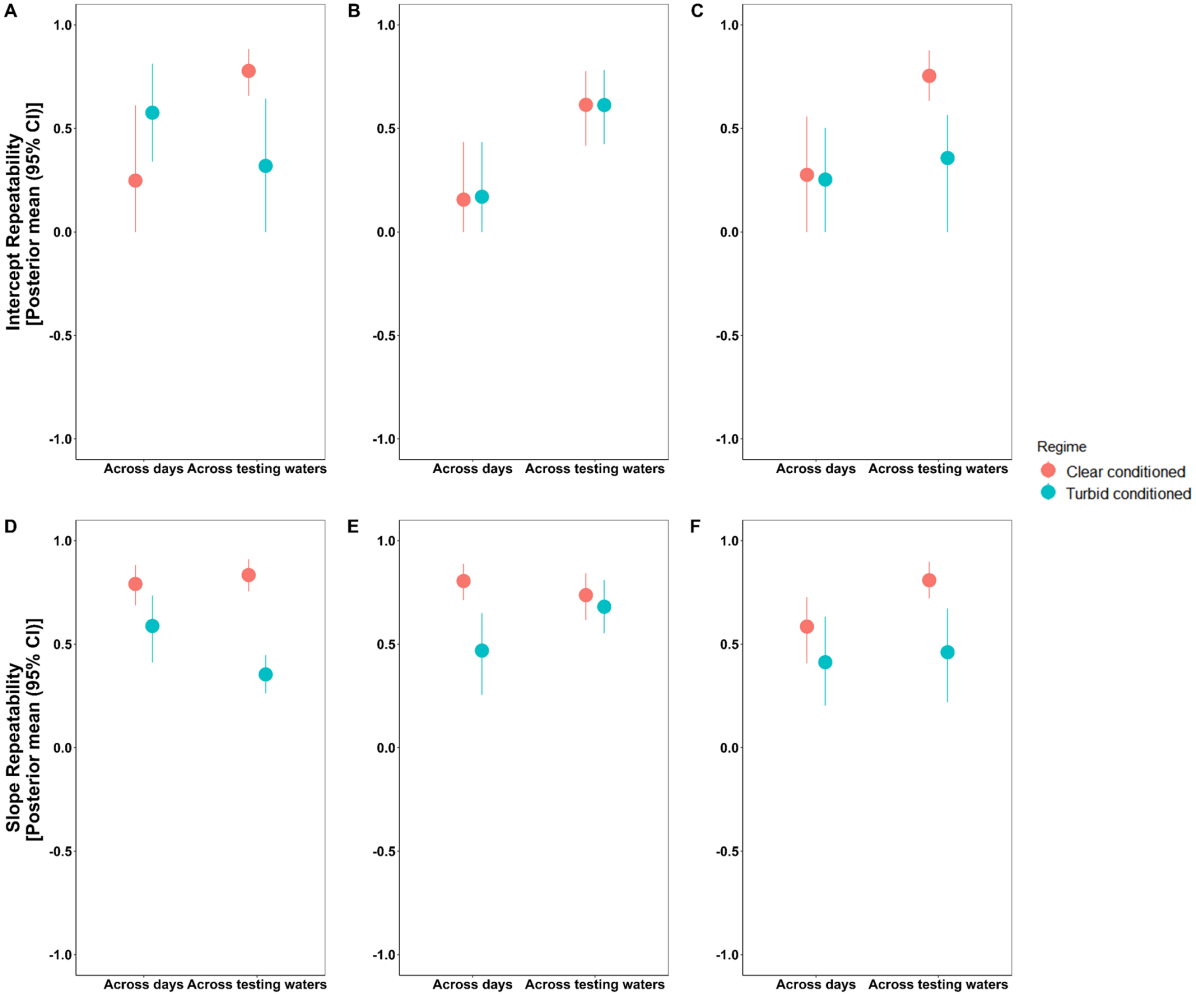
**Intercept- and slope-repeatability estimates plotted for activity, aggression and boldness across trial days and testing waters for conditioning regimes.** Conditioning regimes are color-coded with each dot in the graph representing posterior mean and the corresponding line bars being the spanning 95% credible intervals. Panels A and D represent intercept- and slope-repeatability estimates for activity; B and E are for aggression and; C and F are for boldness.

Conditioning affected individual consistency (intercept-repeatability) across contexts. Like our expectation, individuals conditioned in turbid waters [R=0.576 (0.340, 0.813)] exhibited high intercept repeatability in activity than clear-conditioned [R=0.248 (0.000, 0.612)] when analyzed across repeated trials, whereas they exhibited [R=0.319 (0.000, 0.643)] low repeatability when analyzed across testing waters. Similarly for boldness, turbid-conditioned individuals [R=0.357 (0.000, 0.565)] showed lower repeatability than clear-conditioned [R=0.754 (0.634, 0.877)] across testing waters (Fig. 5). This suggests that prior turbidity exposure increases flexibility within individuals when they face abrupt environmental changes from clear to turbid waters. Aggression showed lower repeatability overall and was less sensitive to conditioning, indicating greater stability across clarity contexts among regimes.

Slope-repeatability estimates also reveal an effect of turbidity (conditioning) treatment on the consistency among individuals' plastic response. Overall, turbid-conditioned individuals showed lower slope repeatability across all three traits compared to those conditioned in clear water, meaning they varied more widely in how they responded to environmental changes indicating less constrained behavioral adjustments (Fig. 5; Table S2). Clear-conditioned individuals showed higher slope repeatability, suggesting more predictable plasticity within individuals (Fig. 5; Table S2).

Change in trait variances was dependent on the repeated trials and testing waters and differed across conditioning treatments. We assess this using random-effects (Tables S3,S4) and residuals intercept-slope (Tables S5,S6) correlations across traits. Across repeated trials, activity intercept-slope correlation was negative [r=−0.567 (−0.881, −0.236)] (Fig. S1) among turbid-conditioned. On the other hand, intercept-slope correlations were negative for activity [r=−0.678 (−0.893, −0.447)], aggression [r=−0.683 (−0.887, −0.451)] (Fig. S2) and boldness [r=−0.866 (−0.972, −0.736)] (Fig. S3) across testing waters among clear-conditioned, suggesting that individuals with higher trait values showed smaller adjustments across environments. Contrary to this, residual intercept-slope correlations indicate that individuals with high activity or aggression or boldness exhibit more change (evident from positive correlations; however, the direction of change is mostly negative) in their trait value across repeated trials or testing waters (Tables S5,S6). This pattern did not differ between conditioning treatments, while the correlation coefficients differed trait-wise across conditioning regimes.

For cross-behavior intercept-slope correlations for among-individuals, the activity and boldness-slope were positively correlated [r=0.376 (0.052, 0.703)] across testing waters in clear water treatment (Fig. S1, Table S4). In contrast, within-individual cross correlations emerged among boldness and aggression-slope [r=0.260 (0.099, 0.444)], and with aggression and boldness-slope [r=0.254 (0.075, 0.432)] in turbid water treatment across trial days (Table S5) as well as across testing waters (Table S6).

### Behavioral syndromes and correlated plasticities

Among-individual trait correlations or behavioral syndromes (Dingemanse et al., 2012), were present for activity and boldness across testing waters among clear-conditioned individuals [r=−0.379 (–0.719, −0.067)] (Table S3,S4). This reflects a stable axis of variation linking exploration and risk-taking.

**Fig. 6.**
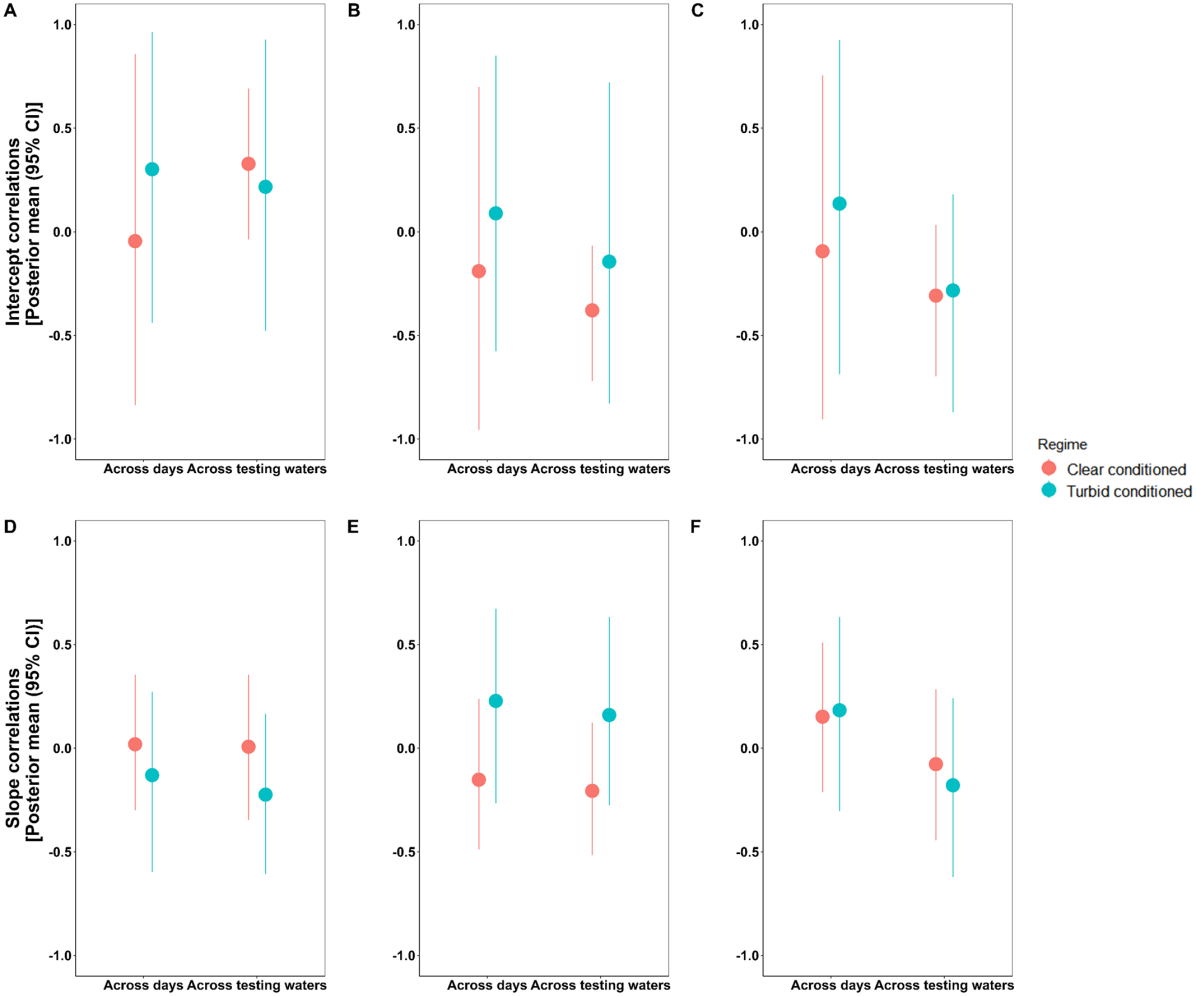
**Behavioral syndromes and correlated plasticities for among-individuals between activity, aggression and boldness represented as intercept- and slope-correlation, respectively.** Conditioning regimes are color-coded with each dot in the graph represents posterior mean and the corresponding line bars represent the spanning 95% credible intervals. Panels A and D represent intercept- and slope-correlations between activity and aggression; similarly, B and E represent correlations between activity and boldness and; C and F represent correlations between aggression and boldness.

Conversely, within-individual trait correlations emerge in turbid-conditioned treatment across trial days such that aggression and boldness are positively correlated [r=0.264 (0.097, 0.428)], and aggression-slope and boldness-slope [r=0.252 (0.074, 0.441)] are positively correlated (Table S5,S6). Similar patterns emerge across testing waters in turbid-conditioned regime, indicating a substantial positive link within-individuals for the traits, aggression and boldness. Whereas in clear-conditioned, activity and boldness [r=−0.188 (−0.336, −0.021)] was negatively correlated across testing waters (Fig. 6). Despite substantial individual variation in plasticity, we found no evidence for among-individual correlated plasticities, suggesting that plastic responses across traits are not jointly regulated.

## DISCUSSION

It is increasingly evident that in addition to mean expression of behaviors, predictions about the behavioral adaptations under changing environment need to include among- and within-individual variations to provide more realistic models of how animals respond. Studies show that environmental gradients can reshape the predictability and integration of behavioral traits (Hewes et al., 2017; Cornwell et al., 2019; Salazar et al., 2023; Mitchell et al., 2024). Yet, empirical approaches that evaluate mean responses together with among- and within-individual (co)variances and plasticities remain rare, despite their relevance for understanding how behavioral phenotypes evolve in dynamic environments (Mitchell and Houslay, 2021; Sheehy and Laskowski, 2023). Our study contributes to this integration by showing how prolonged changes in water clarity can shape short-term adjustments in zebrafish coping behaviors through effects on means, variance components, and the coordination of behavioral correlations and plasticity.

We discovered that a month-long exposure to turbidity did not alter the mean expression in activity, aggression, or boldness between conditioned groups; instead, the shift in mean expression was in response to immediate change in turbidity as well as over repeated trials, consistent with turbidity acting as a contextual rather than developmental driver for adult zebrafish. This aligns with earlier work demonstrating rapid, reversible effects of turbidity on foraging, movement, and risk-taking (Gray et al., 2012a; Suriyampola et al., 2018), but little evidence for long-lasting shifts in baseline behaviors in adults. We, however, cannot completely negate this shift in mean being due to habituation effect, not accounted in our study. To minimize habituation effects, individuals were tested with a 2-day gap between trials. Therefore, we expect habituation effect to be more pronounced within trials conducted on the same day (across different clarity conditions) than for across different days. Nevertheless, taken together, all three traits showed an effective interaction between test days (trial repeats) and testing waters (water clarity), further supporting the idea that turbidity may influence behavioral means, but primarily as a short-term effect.

Sex differences for activity were observed and an interaction between size and sex indicated that slightly larger females tended to be more active. Since there was no interaction effect between conditioning treatment and sex, we did not partition trait variances further between females and males or pursue sex-differences beyond the mean expression. This does limit our study regarding the understanding of trait integration differences between females and males. Although evidence for sex-differences in trait (co)variances in risk-taking behaviors is limited, we expected such differences in zebrafish due to their differences in mating styles (Spence et al., 2008).

Where conditioning did matter was in the structure of individual differences. Turbid-conditioned individuals were more flexible – exhibiting reduced repeatability across testing waters – suggesting that long-term exposure to turbidity promotes behavioral lability. In contrast, clear-conditioned individuals showed higher intercept- and slope-repeatability, indicative of more predictable coping strategies. These patterns mirror theoretical expectations from coping-style frameworks: reactive phenotypes are thought to be more plastic and flexible under stress, whereas proactive phenotypes are more rigid (Koolhaas et al., 1999; Øverli et al., 2007). Here, prolonged turbidity exposure might function as a persistent stressor that promotes reactive-like, flexible responses. Interestingly, aggression was largely unaffected by either conditioning or contextual turbidity, hinting that not all traits are equally sensitive to sensory environmental change. This contrasts with boldness and activity, which are more tightly linked to risk assessment and visually guided decision-making. However, we suggest caution in the interpretation of our results since some of our intercept-repeatability estimates were marginally overlapping zero in their lower confidence intervals (CI) and the contrast between conditioning regimes is interpreted through direct inspection of repeatability values (Fig. 5). Also, our study is not well sampled at within-individual level, i.e. two repeats per gradient per individual – for labile traits, two trials per context may not capture the correct rank order differences (Biro, 2012) and hence, our patterns may change with the incorporation of more trials per individual. While future studies could increase replicates per individual, we chose this design to minimize habituation and maintain manageable trial stress levels. At this point, it is also difficult to determine whether the order in which behaviors were tested (see details of experimental protocol in the Materials and Methods) influenced overall trait consistency. We assumed that any carry-over effects from previous behavioral tests would be consistent across individuals, allowing us to reasonably disregard this constraint (Bell, 2013). Additionally, to minimize the carry-over effects, a 2-min acclimation period was provided between behavioral tests (see Materials and Methods).

Behavioral syndromes are traditionally viewed as stable axes of trait covariation (Sih et al., 2004), but recent work suggests that syndromes may be highly context dependent and shaped by environmental plasticity (Mitchell & Biro, 2017; Mitchell et al., 2024). Our study provides support for this environmental dependence. We found only one among-individual behavioral syndrome: a negative activity-boldness correlation, but only in clear-conditioned fish. This resembles exploration-risk-taking axes described in many taxa (Kanda et al., 2012; Herde and Eccard, 2013; Garamszegi et al., 2013; Fu et al., 2021; Axling et al., 2023) and suggests that consistent trait integration emerges more readily under conducive environments, where behavioral decisions are less constrained by uncertainty. In contrast, turbid-conditioned fish showed trait integration primarily at the within-individual level, with coordinated adjustments between aggression and boldness across both days and water clarities. Stronger within-individual covariation suggests that turbidity can synchronize short-term behavioral adjustments rather than stabilizing long-term trait correlations, supporting the idea that environmental stress can temporarily align behavioral responses even when among-individual syndromes are weak. However, our design is not enough to comment on the lasting effect of this alteration. Since our design included an arbitrary period of conditioning, i.e. 1 month, this raises questions about the duration of conditioning for which its effects on trait integration may become pronounced. Although our focus was on adult conditioning effects rather than duration dependence, ideally, our study could be followed up with an experimental design incorporating conditioning versus no conditioning along with longer duration of conditioning.

Despite substantial variation in plasticity values, we found no among-individual correlated plasticities across traits. This implies that individuals did not consistently vary in how plastic they were across multiple traits, a result consistent with other studies that report weak or absent correlations in plasticity across traits (Cornwell et al., 2019; Salazar et al., 2023). However, within-individual correlated plasticities were present, and their structure differed across conditioning regimes. In turbid-conditioned fish, aggression and boldness shifted together across environments. In clear-conditioned fish, activity and boldness correlated within individuals, but their plasticities did not. These contrasting patterns emphasize that environmental experience influences not only baseline behavior but also the architecture of plastic responses, adding nuance to current theories of plasticity integration (Stamps, 2016; Sheehy and Laskowski, 2023).

Most models of personality development emphasize that stable among-individual variation often originates early in life (Groothuis and Trillmich, 2011). And these experiences are contingent on genetic background of individuals. Adult experiences may modulate behavioral expression but rarely reorganize trait (co)variances unless environmental exposure is strong or prolonged. Our month-long conditioning period produced detectable effects on variance structure but not on the full architecture of trait integration. This suggests that adult conditioning may influence behavioral flexibility but may not fully canalize new behavioral syndromes. Given that turbidity naturally fluctuates in zebrafish habitats and larvae experience similar clarity regimes as adults (Spence et al., 2008), adults' response could be a cumulative one. Therefore, future work should incorporate developmental manipulations to test whether chronic early-life turbidity produces deeper changes to integrated behavioral phenotypes.

As sedimentation and turbidity rise due to anthropogenic pressures (Scheurer et al., 2009; Wong and Candolin, 2014), populations will increasingly navigate sensory-degraded habitats. Our results highlight several important implications: individual variation in plasticity is substantial, suggesting some individuals may cope better with environmental uncertainty. Trait integration changes with sensory conditions, meaning that ecological interactions (predation, foraging, social behavior) may shift in unpredictable ways as turbidity increases. Long-term turbidity promotes behavioral flexibility, potentially buffering populations against rapid change but also weakening trait predictability and syndromes that may be ecologically functional. Together, these findings underscore that environmental temporal heterogeneity restructures behavioral integration across multiple hierarchical levels, providing a richer understanding of how populations may persist or fail to persist under accelerating environmental change.

While our study advances understanding of how adult experiences shape behavioral syndromes and correlated behavioral plasticities, several questions remain open, for example, under what circumstances are behavioral syndromes and correlated behavioral plasticities linked? How can ontogeny stabilize this link? Although our results point in promising direction, their broader applicability needs to be evaluated through rigorous study-designs and well-developed theoretical frameworks.

## MATERIALS AND METHODS

### Study subjects and housing of population

Adult zebrafish were collected during the pre-monsoon season from low-flowing native habitats in Howrah, West Bengal India, where both juveniles and adults co-occur. Caught by a local fisherman in groups numbering approximately 220-250 fish per catchment, fish were supplied to the laboratory in July 2021. Before conducting the experiments, stock fish were housed and acclimated for 30 days to the lab conditions, maintained at an ambient temperature of 23±3°C in the natural light:dark cycle, in large aerated, bare-glass tanks of size 45 cm×24 cm×30 cm (with filtered water level up to 15 cm) in a mixed-sex ratio (∼100 females and 100 males). Sexing was performed by external morphology (gravid abdomen in females, flat in males) (Spence et al., 2008). Loose or reconstituted freeze-dried blood worms were given as food *ad libitum* once per day.

### Experimental protocol

A randomly selected subset of the stock population was divided into four sub-groups and kept separately in four aerated tanks. Two sub-groups were conditioned in clear water (CW) and the other two in turbid water (TW) for 30 days. After conditioning, we isolated individuals from their respective conditioning regime into separate cylindrical, transparent plastic jars (one individual per jar), which were marked to maintain the identity of the individual and to enable tracking individual behaviors (Roy and Bhat, 2018a). Each individual was then tested in both CW and TW on the same day and tested again in CW and TW after a gap of 2 days.

#### Conditioning

We randomly selected 100 individuals from the stock and transferred them into four half-filled, aerated (using air stones) glass tanks (dimensions: 45 cm×24 cm×30 cm), with each tank holding about 25 individuals with ∼1:1 sex ratio. We used extra pure Kaolin-clay powder (LOBA-CHEMIE) (Borner et al., 2015; Sekhar et al., 2019) to make the water turbid. Kaolin clay particles have large surface areas, keeping the particles suspended in water for a longer period (Meager et al., 2005). Based on the spectrophotometric analysis of water samples from the natural habitats of zebrafish, we used 1.18 m^−1^ and 5.08 m^−1^ of beam attenuation values for CW and TW tanks, respectively, during conditioning. However, Kaolin particles settle down under the influence of gravity, hence decreasing the turbidity of the system if the system is kept undisturbed for a longer period. Using air-stones was not enough to maintain prolonged turbidity; therefore, we adopted a mechanical method of churning the water to keep relatively consistent levels of turbidity in conditioning tanks. The water of all CW and TW tanks was churned with five back-and-forth strokes across the length of the tank using a fishnet, four times a day (06:00, 10:00, 14:00 and 18:00) for 30 days. However, we acknowledge that manual churning by the experimenter can itself induce fear response among individuals.

After the end of the conditioning period, individuals were isolated in transparent-cylindrical jars (∼1 l volume), each filled with 500 ml of clear water, to maintain their identity during the behavior experiments. Complete isolation can generate stress-induced responses in behavior (Daniel and Bhat, 2022b). To minimize such effects, we used transparent jars that aided visual contact between individuals and added an extra of 100 ml of water from the conditioning tank to each of the 500 ml filled jars to facilitate social-chemical cues. Each day, the position of the jars was randomized until the end of the experiment to avoid any possible copying of behaviors by neighboring fish.

#### Behavioral tests

**Fig. 7.**
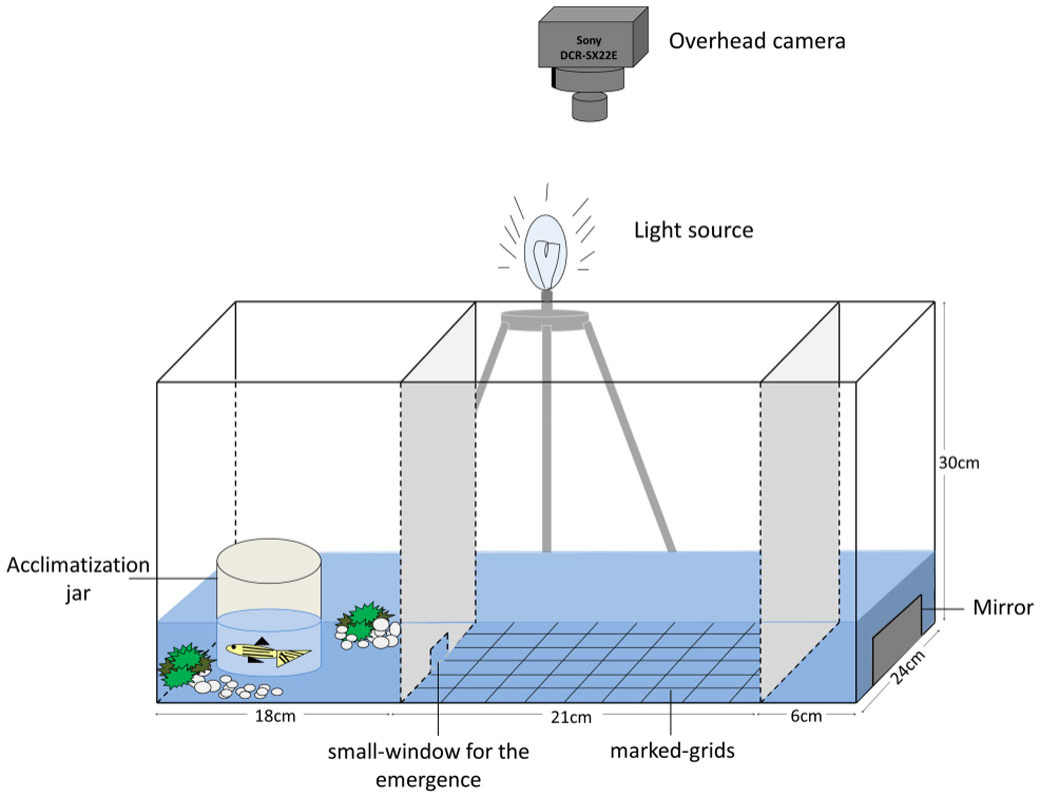
**An illustration of experimental set-up.** Schematic view of the experimental set-up (lateral-view) used for clear water and turbid water trials, with three separate chambers for testing boldness, activity and aggression.

At the end of the conditioning period, we tested each individual from CW and TW groups for their behavioral traits (i.e. activity, aggression, and boldness) in an experimental arena set-up, as shown in Fig. 7. While these behaviors inherently represent stress-related behavioral responses, other physiological correlates such as cortisol-levels, etc., have also been established as direct measures of stress-responses among animals (Archard et al., 2012; Killen et al., 2013). For our study, we relied on behavioral responses as direct measures of stress mitigation among fish. Each individual in the CW regime was first tested in CW and then in TW on the same day. Similarly, an individual conditioned in TW was first tested in TW and then in CW. The testing order was fixed (as mentioned above) in both regimes to control for between-individual error variance, which would have increased due to differences in carryovers (if any) if the individuals were tested in a randomized order (Bell, 2013). The level of turbidity during the behavioral tests was kept the same as was used during conditioning. We repeated the behavioral measurements on the individuals after a gap of 2 days (day 1 trials and day 2 trials) in both CW and TW to test for consistency.

The experimental arena set-up (dimensions: 45 cm×24 cm×30 cm) (Chen et al., 2018) was partitioned into three chambers as shown in Fig. 7.

#### Boldness (refuse-emergence test)

The first chamber (18 cm×24 cm) was covered in artificial plants and marbles on one side while keeping a transparent acclimatization jar on the other side. A small window (3 cm×3 cm) carved at the bottom of the partition separating the first chamber with the rest of the tank, allowed individuals to escape the complex environment into the second chamber. We measured boldness as the amount of time an individual took to come out of the first chamber through the small window (Brown and Braithwaite, 2004; Réale et al., 2007). Individuals were assigned boldness scores equal to the latency to emerge from the complex chamber over a 5-min trial. Since each trial lasted for 300 s, the highest boldness score that could be assigned was 300 while the lowest boldness score was 0. Here, the boldness score is inverse of the actual boldness of the fish. This implies that individuals with lower boldness scores than the average individual were considered bold, while individuals with higher boldness scores than the average individual were considered as shy.

#### Activity (open-field test)

The second chamber (21 cm×24 cm) was an open-field (Réale et al., 2007), marked with 56 square-grids (3 cm×3 cm) at the bottom. We measured the activity of individuals by manually noting the position of the fish each second during its swim from the video recordings. The activity scores for individuals were calculated as the number of unique-square grids crossed in the first and second minutes. We treated each minute of the trial as independent of the other; therefore, the unique grids that overlapped between the first and second minute were counted twice. Since the total number of square-grids was 56, the highest activity score that could be assigned was 112 (56+56), while the lowest activity score, in the case where the subject fish is frozen, could be 2 (1+1). Individuals with higher activity scores than the average individual were considered active, while individuals with lower activity scores than the average individual were considered less active.

#### Aggression (mirror test)

The third chamber (6 cm×24 cm) had a mirror kept behind the tank wall that covered about ∼90% of the area of the wall. We measured aggression as the number of times an individual ‘attacks’, i.e. hits at the mirror with its snout (Way et al., 2015). Each individual was assigned an aggression score equal to the total number of attacks on the mirror in a 2-min trial. Individuals with higher aggression scores than the average individual were considered aggressive, while individuals with lower aggression scores than the average individual were considered less aggressive.

For each trial, a random individual (‘test’) fish was picked by gently netting it from its respective cylindrical jar and transferred into the acclimatization jar (a transparent cylindrical container, open at both ends, placed on one-side of the experimental arena), where it was allowed to acclimatize for 2 min. The acclimatization jar was then lifted gently, and the fish was allowed to swim and come out of the chamber through the small window in the next 5 min. Once the fish came out of the chamber, the window was covered with a lid to stop the fish from going back to the first chamber. Fish that did not emerge within 5 min were gently netted and put in the second chamber. Following this, the fish were allowed to swim in the open field for about 4 min and the number of unique grids crossed during the last 2 min was noted as the activity score. Following this, the partition separating the mirror from view was gently removed and the fish was allowed to swim around and see and attack the mirror for the next 3 min, and the number of attacks the individual made over 2 min was noted.

We used two experimental arenas of the same dimensions simultaneously to perform behavior tests in both clear and turbid waters. The water was churned between each trial in both tanks to maintain similar levels of turbidity across all the trials. Each trial was recorded with a video camera (Sony DCR-SX22E and Sony DCR-PJ5E) placed vertically above the arena. Individuals who did not respond during the first trial did respond in the second trial. So, we included all the individuals in the analyses. Sizing was done once the trials were over. Three females and five males died in each regime during the conditioning, so the resulting sample size was 42 (24 females and 18 males) in CW regime and 42 (22 females and 20 males) in TW regime. Mortality was likely random, due to natural reasons or individual differences in physical condition.

### Statistical analysis

#### General statistical procedures

All analyses were carried out in R programming platform (v.4.4.2, R Core Team, 2025) run through RStudio v.12.0. We used an R-package, *MCMCglmm* (Hadfield, 2010), that uses Bayesian framework applied through Markov chain Monte Carlo (MCMC) methods to fit our multivariate random regression models. Activity, aggression, and boldness scores were z-transformed to avoid large variance differences in the covariance matrices. All three traits were modeled using ‘family’ of ‘gaussian’ distributions (Hadfield, 2019). We used weakly-informed parameter-expanded priors (with belief parameter, nu=3; prior-mean, alpha.mu=0; and covariance matrix, alpha.V=1000) for random effects and for the residuals (with belief parameter, nu=3) (Houslay and Wilson, 2017). The model was iterated over 10,000,000 times with an initial burn of 1,000,000. Each chain was thinned and sampled at an interval of 10,000, resulting in a low autocorrelation (<0.025) between thinned samples. We estimated posterior means and 95% CI (highest posterior density) across thinned samples for the fixed effects, random effects, residuals and covariances. For the assessment of a possible treatment effect, we examined whether CI overlap with zero, indicating that the effect does not differ from zero (Hadfield, 2010; Dingemanse and Dochtermann, 2013). We also interpreted treatment effects for the fixed effects using *P*-values with 0.05 as the level of significance.

#### Estimating mean-effects

We estimated the fixed effects for each trait for the following factors: trial day (with levels, day 1 and day 2), testing water conditions (with levels, CW and TW) and conditioning regime (with levels, clear-water and turbid-water), and the interaction between them. We also included size and sex as fixed factors and the interaction between them. Since there was a high ceiling effect in our boldness scores (300s; in 45.6% of observations) and such individuals were gently netted to the activity arena, we addressed this using ‘nudged’ as a fixed factor (with levels, yes and no). Additionally, we tried to include other factors such as habituation (in terms of the order of testing in waters), tank identity (as the social environment, two for each conditioning regime) and interaction between nudging and conditioning, but our models failed to initialize with them and hence, were dropped from analysis.

#### Estimating random intercepts and random slopes

Individual identities were included as the random effect to estimate random intercepts and random slopes for traits, and random intercepts and random slopes were specified for residuals also. In our random slopes, we fitted trial days and testing waters separately, allowing us to estimate (co)variation due to endogenous and contextual plasticity, respectively. While our (co)variances were partitioned by conditioning regimes, trait (co)variances were assessed using unstructured variance-covariance matrix (here, a 6×6 matrix) that allowed us to estimate (co)variances of all combinations between intercepts and slopes for both random effects and residuals across the three traits. Hence, for each regime (clear-water conditioned and turbid-water conditioned), there were four 6×6 matrices: two for the random effects with random slopes varying by trial days and testing waters while the other two for their respective residuals.

#### Estimating variance ratios and correlations

Since our model allowed us to estimate random intercepts and random slopes for residuals as well, we used repeatability as a standardized metric for consistent variation in each trait as well as consistency in their plasticity, defined as the ratio of among-individual variance (random effect variance, V_ind_) over the sum of V_ind_ and within-individual variance (residual variance, V_c_) (Boake, 1989; Nakagawa and Schielzeth, 2010). Intercept-slope correlations were calculated by dividing the intercept-slope covariance with the product of square-roots of intercept and slope variance. Similarly, phenotypic correlations between traits were calculated by dividing trait covariances with the product of square-roots of each trait's variance, for the assessment of trait integration across conditioning regimes.

### Ethics statement

Guidelines outlined by the Committee for the Purpose of Control and Supervision of Experiments on Animals (CPCSEA), Ministry of Fisheries, Animal Husbandry and Dairying, Government of India were followed in all aspects of maintenance and experimentation. All experimental protocols followed here have been approved by the Institutional Animal Ethics Committee's (IAEC) and guidelines of Indian Institute of Science Education and Research (IISER) Kolkata, Government of India (Approval number IISERK/IAEC/AP/2021/70).

## Supplementary Material

10.1242/biolopen.062341_sup1 Supplementary information
