# Supplementary material for: Behavioral trait (co)variances and plasticity in response to turbidity in wild zebrafish (Danio rerio)
Source: Biol Open. 2026 Feb 24;15(2):bio062341. doi: 10.1242/bio.062341 (PMC12969766; doi:10.1242/bio.062341)
Supplement: Supplementary information [file biolopen-15-062341-s1.pdf]

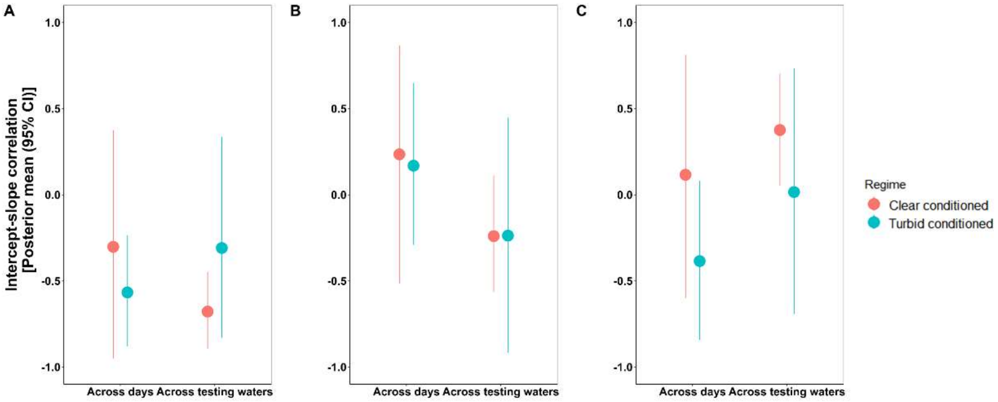

Fig. S1. Among-individuals intercept-slope correlations in activity with activity- slope (A), aggression-slope (B) and boldness-slope (C) across trial days and testing waters. Conditioning regimes are color-coded with each dot in the graph representing posterior mean and the corresponding line bars being the spanning 95% credible intervals.

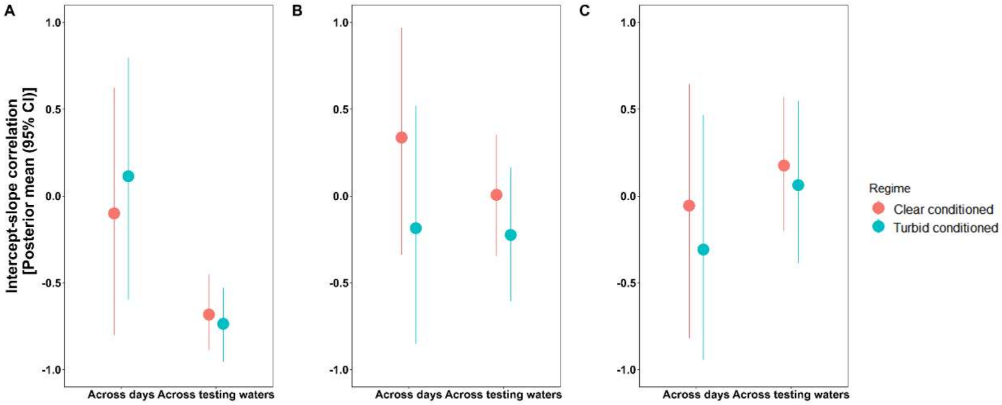

Fig. S2. Among-individuals intercept-slope correlations in aggression with aggression-slope (A), activity-slope (B) and boldness-slope (C) across trial days and testing waters. Conditioning regimes are color-coded with each dot in the graph representing posterior mean and the corresponding line bars being the spanning 95% credible intervals.

Biology Open • Supplementary information

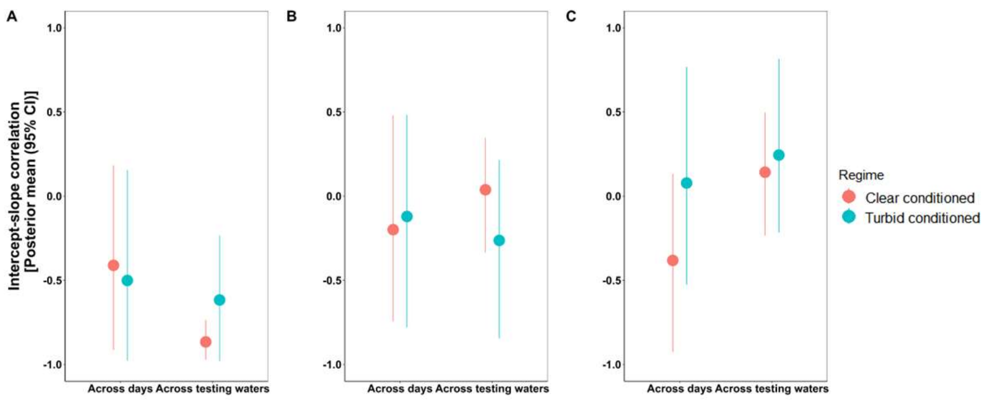

Fig. S3. Among-individuals intercept-slope correlations in boldness with boldness- slope (A), activity-slope (B) and aggression-slope (C) across trial days and testing waters. Conditioning regimes are color-coded with each dot in the graph representing posterior mean and the corresponding line bars being the spanning 95% credible intervals.

Table S1. Intercept-repeatability estimates represented by the posterior mean with 95% credible intervals; for clear-conditioned and turbid-conditioned regimes across three traits. The estimates are reported across trial days and across testing waters.

	Activity	Aggression	Boldness
	Clear water conditioned regime		
Across trial days	0.248<br>(0, 0.612)	0.156<br>(0, 0.435)	0.276<br>(0, 0.557)
Across testing waters	0.778<br>(0.657, 0.884)	0.614<br>(0.417, 0.776)	0.754<br>(0.634, 0.877)
	Turbid water conditioned regime		
Across trial days	0.576<br>(0.340, 0.813)	0.170<br>(0, 0.434)	0.253<br>(0, 0.503)
Across testing waters	0.319<br>(0, 0.643)	0.613<br>(0.424, 0.782)	0.357<br>(0, 0.565)

Table S2. Slope-repeatability estimates represented by the posterior mean with 95% credible intervals; for clear-conditioned and turbid-conditioned regimes across three traits. The estimates are reported across trial days and across testing waters.

	Activity	Aggression	Boldness
	Clear water conditioned regime		
Across trial days	0.791<br>(0.688, 0.882)	0.805<br>(0.713, 0.888)	0.585<br>(0.408, 0.727)
Across testing waters	0.834<br>(0.756, 0.910)	0.737<br>(0.616, 0.842)	0.809<br>(0.721, 0.898)
	Turbid water conditioned regime		
Across trial days	0.588<br>(0.411, 0.735)	0.469<br>(0.255, 0.651)	0.413<br>(0.204, 0.632)
Across testing waters	0.354<br>(0.262, 0.447)	0.681<br>(0.553, 0.809)	0.461<br>(0.220, 0.672)

Biology Open • Supplementary information

Table S3. Reported are the posterior means and 95% credible intervals for among-individuals correlation coefficients for all pairs of intercepts, slopes and intercept-slope across the trial days. Boldfaced estimates represent the ones where the credible intervals did not overlap zero, indicating a possible effect.

	Activity (int)	Activity (Trial Day)	Aggression (int)	Aggression (Trial Day)	Boldness (int)	Boldness (Trial Day)
	Clear water conditioned regime					
Activity (int)		-0.302 (-0.949, 0.376)	-0.045 (-0.836, 0.858)	0.235 (-0.514, 0.868)	-0.190 (-0.956, 0.699)	0.116 (-0.599, 0.813)
Activity (Trial Day)			0.337 (-0.339, 0.969)	0.019 (-0.299, 0.354)	-0.199 (-0.744, 0.479)	-0.152 (-0.488, 0.238)
Aggression (int)				-0.100 (-0.801, 0.624)	-0.094 (-0.904, 0.755)	-0.055 (-0.820, 0.645)
Aggression (Trial Day)					-0.382 (-0.926, 0.132)	0.152 (-0.212, 0.509)
Boldness (int)						-0.411 (-0.915, 0.183)
Boldness (Trial Day)						
	Turbid water conditioned regime					
Activity (int)		**-0.567** (-0.881, -0.236)	0.302 (-0.440, 0.963)	0.169 (-0.291, 0.647)	0.089 (-0.577, 0.850)	-0.385 (-0.843, 0.081)
Activity (Trial Day)			-0.185 (-0.852, 0.521)	-0.130 (-0.598, 0.272)	-0.121 (-0.780, 0.483)	0.228 (-0.266, 0.673)
Aggression (int)				0.114 (-0.597, 0.798)	0.136 (-0.688, 0.925)	-0.308 (-0.943, 0.468)
Aggression (Trial Day)					0.078 (-0.526, 0.768)	0.183 (-0.304, 0.632)
Boldness (int)						-0.501 (-0.977, 0.155)
Boldness (Trial Day)						

Biology Open • Supplementary information

Table S4. Reported are the posterior means and 95% credible intervals for among-individuals correlation coefficients for all pairs of intercepts, slopes and intercept-slope across the testing waters. Boldfaced estimates represent the ones where the credible intervals did not overlap zero, indicating a possible effect.

	Activity (int)	Activity (Testing Water)	Aggression (int)	Aggression (Testing Water)	Boldness (int)	Boldness (Testing Water)
	Clear water conditioned regime					
Activity (int)		**-0.678** (-0.893, -0.447)	0.328 (-0.037, 0.691)	-0.240 (-0.565, 0.113)	**-0.379** (-0.719, -0.067)	**0.376** (0.052, 0.703)
Activity (Testing Water)			0.117 (-0.252, 0.493)	0.007 (-0.346, 0.354)	0.038 (-0.336, 0.347)	-0.206 (-0.516, 0.123)
Aggression (int)				**-0.683** (-0.887, -0.451)	-0.308 (-0.698, 0.034)	0.176 (-0.201, 0.571)
Aggression (Testing Water)					0.142 (-0.234, 0.497)	-0.077 (-0.444, 0.284)
Boldness (int)						**-0.866** (-0.972, -0.736)
Boldness (Testing Water)						
	Turbid water conditioned regime					
Activity (int)		-0.309 (-0.831, 0.335)	0.217 (-0.478, 0.927)	-0.237 (-0.916, 0.449)	-0.144 (-0.828, 0.721)	0.016 (-0.692, 0.734)
Activity (Testing Water)			0.282 (-0.153, 0.687)	-0.224 (-0.607, 0.165)	-0.263 (-0.844, 0.215)	0.160 (-0.275, 0.632)
Aggression (int)				**-0.736** (-0.954, -0.528)	-0.283 (-0.870, 0.181)	0.063 (-0.386, 0.548)
Aggression (Testing Water)					0.244 (-0.216, 0.815)	-0.179 (-0.622, 0.241)
Boldness (int)						**-0.617** (-0.980, -0.233)
Boldness (Testing Water)						

Table S5. Reported are the posterior means and 95% credible intervals for within-individuals correlation coefficients for all pairs of intercepts, slopes and intercept-slope across the trial days. Boldfaced estimates represent the ones where the credible intervals did not overlap zero, indicating a possible effect.

	Activity (int)	Activity (Trial Day)	Aggression (int)	Aggression (Trial Day)	Boldness (int)	Boldness (Trial Day)
	Clear water conditioned regime					
Activity (int)		**0.791** (0.715, 0.858)	0.122 (-0.032, 0.297)	0.121 (-0.049, 0.314)	-0.183 (-0.332, 0.008)	-0.174 (-0.369, 0.021)
Activity (Trial Day)			0.116 (-0.065, 0.310)	0.115 (-0.086, 0.318)	-0.177 (-0.338, 0.013)	-0.169 (-0.382, 0.026)
Aggression (int)				**0.829** (0.767, 0.896)	-0.137 (-0.314, 0.017)	-0.131 (-0.343, 0.073)
Aggression (Trial Day)					-0.132 (-0.344, 0.040)	-0.130 (-0.331, 0.072)
Boldness (int)						**0.651** (0.532, 0.770)
Boldness (Trial Day)						
	Turbid water conditioned regime					
Activity (int)		**0.890** (0.848, 0.927)	0.043 (-0.123, 0.217)	0.039 (-0.138, 0.226)	-0.091 (-0.266, 0.063)	-0.094 (-0.265, 0.095)
Activity (Trial Day)			0.042 (-0.158, 0.212)	0.038 (-0.143, 0.245)	-0.093 (-0.279, 0.071)	-0.095 (-0.284, 0.088)
Aggression (int)				**0.894** (0.853, 0.933)	**0.264** (0.097, 0.428)	**0.254** (0.075, 0.432)
Aggression (Trial Day)					**0.260** (0.099, 0.444)	**0.252** (0.074, 0.441)
Boldness (int)						**0.803** (0.727, 0.866)
Boldness (Trial Day)						

Biology Open • Supplementary information

Table S6. Reported are the posterior means and 95% credible intervals for within-individuals correlation coefficients for all pairs of intercepts, slopes and intercept-slope across the testing waters. Boldfaced estimates represent the ones where the credible intervals did not overlap zero, indicating a possible effect.

	Activity (int)	Activity (Testing Water)	Aggression (int)	Aggression (Testing Water)	Boldness (int)	Boldness (Testing Water)
	Clear water conditioned regime					
Activity (int)		**0.791** (0.711, 0.864)	0.123 (-0.032, 0.290)	0.120 (-0.068, 0.327)	**-0.188** (-0.336, -0.021)	-0.179 (-0.391, 0.006)
Activity (Testing Water)			0.119 (-0.071, 0.311)	0.119 (-0.087, 0.338)	**-0.182** (-0.343, -0.002)	-0.175 (-0.365, 0.033)
Aggression (int)				**0.823** (0.752, 0.882)	-0.138 (-0.294, 0.041)	-0.146 (-0.333, 0.048)
Aggression (Testing Water)					-0.140 (-0.312, 0.052)	-0.146 (-0.337, 0.057)
Boldness (int)						**0.658** (0.532, 0.769)
Boldness (Testing Water)						
	Turbid water conditioned regime					
Activity (int)		**0.884** (0.835, 0.928)	0.042 (-0.115, 0.232)	0.031 (-0.167, 0.204)	-0.101 (-0.264, 0.083)	-0.098 (-0.300, 0.088)
Activity (Testing Water)			0.030 (-0.164, 0.204)	0.022 (-0.175, 0.216)	-0.103 (-0.292, 0.074)	-0.103 (-0.298, 0.090)
Aggression (int)				**0.891** (0.849, 0.930)	**0.264** (0.101, 0.414)	**0.252** (0.064, 0.420)
Aggression (Testing Water)					**0.259** (0.091, 0.435)	**0.250** (0.042, 0.431)
Boldness (int)						**0.808** (0.735, 0.875)
Boldness (Testing Water)						

Biology Open • Supplementary information
